# Supplementary figures and images for: Drosophila models of pathogenic copy-number variant genes show global and non-neuronal defects during development
Source: PLoS Genet. 2020 Jun 24;16(6):e1008792. doi: 10.1371/journal.pgen.1008792 (PMC7313740; doi:10.1371/journal.pgen.1008792)

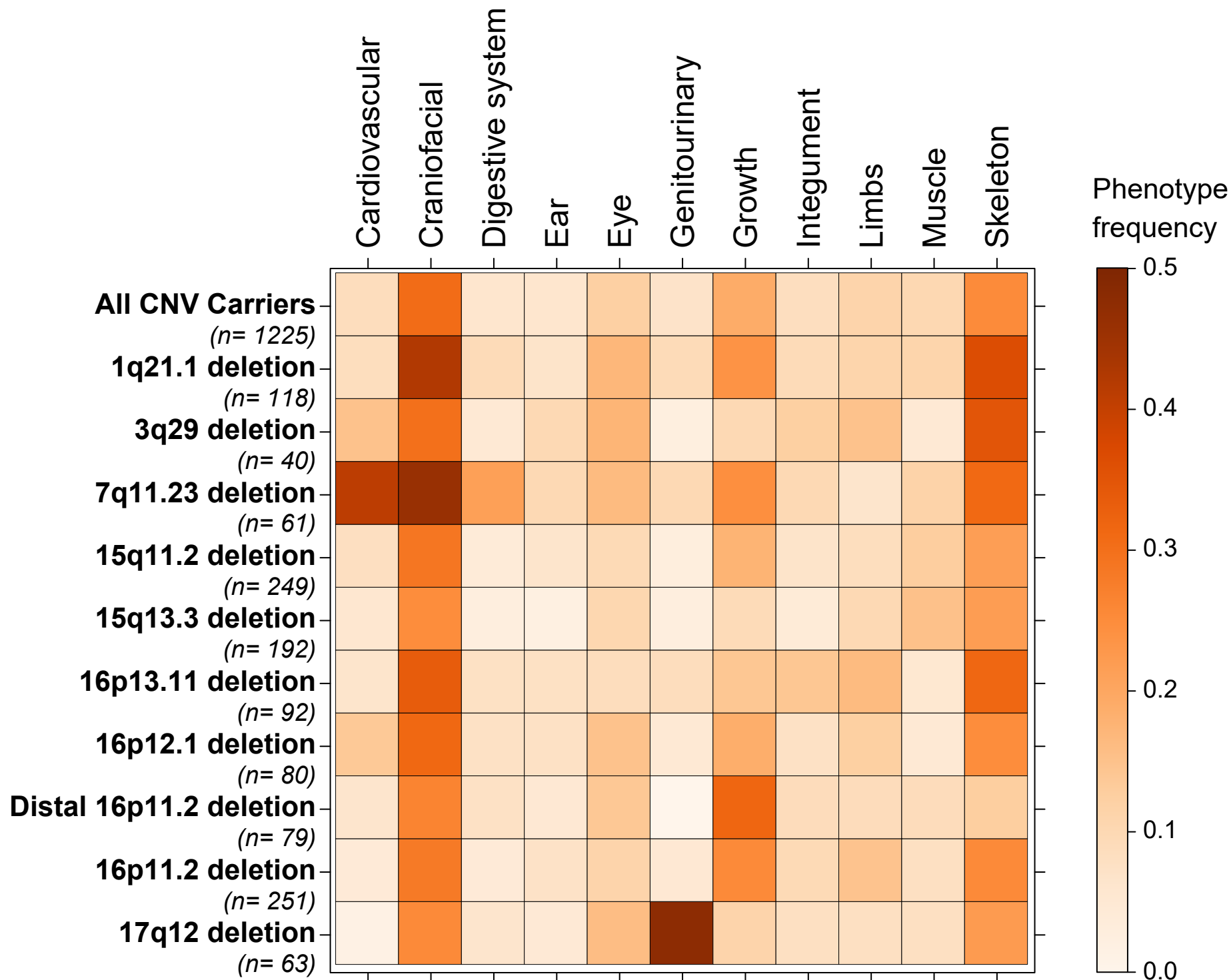

Supplement: S1 Fig — Heatmap shows frequencies of non-neuronal developmental phenotypes observed in 1,225 human carriers of 10 pathogenic CNV deletions, curated from the DECIPHER database. CNV carriers show a variety of phenotypes that manifest across different tissues, including eye, limbs, muscle, and skeleton. (PDF) [file pgen.1008792.s001.pdf]

**A**

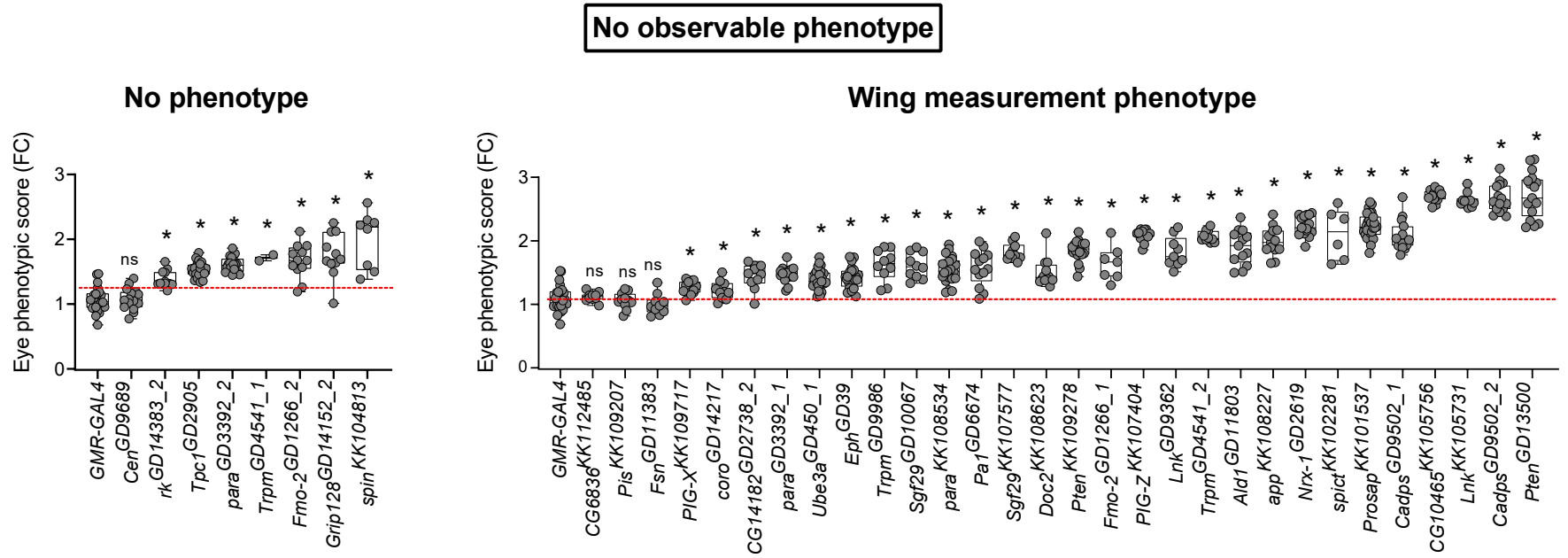

**B**

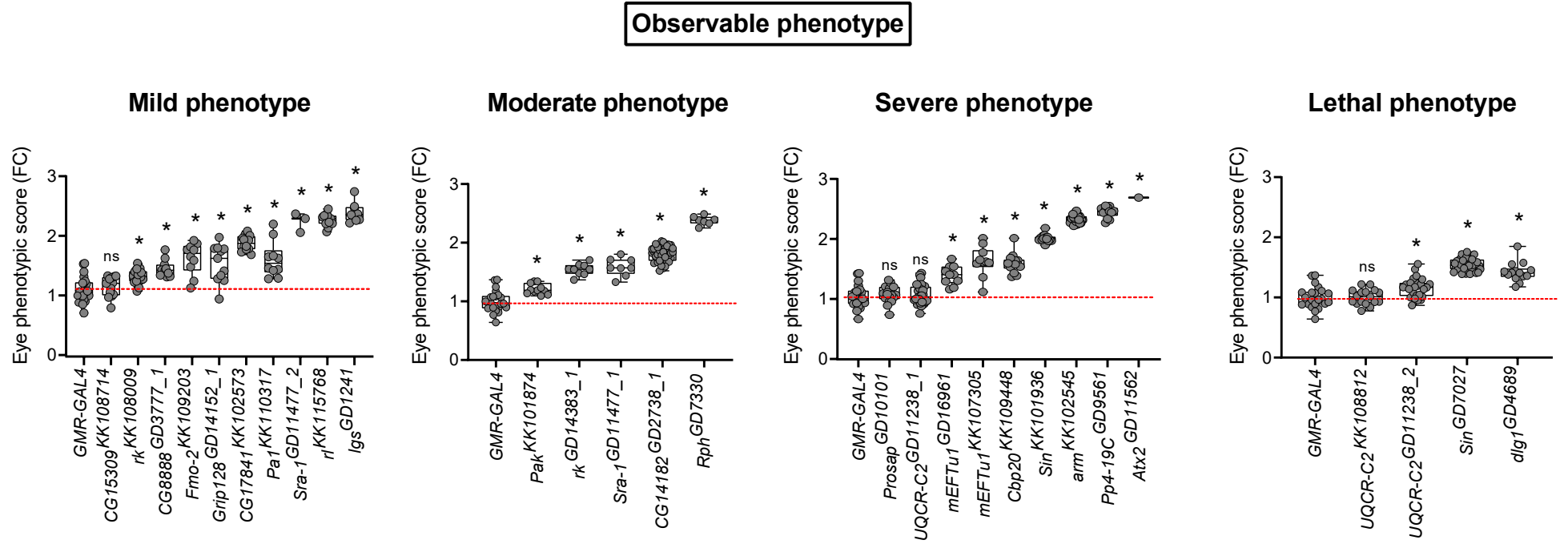

Supplement: S3 Fig — Boxplots show Flynotyper-derived phenotypic scores for 66 tested adult eyes with eye-specific knockdown (GMR-GAL4) of select homologs of CNV and neurodevelopmental genes, normalized as fold-change (FC) to control values (n = 1–40, *p < 0.05, one-tailed Mann–Whitney test with Benjamini-Hochberg correction). RNAi lines that do not show any observable qualitative adult wing phenotypes, including lines that show wing measurement phenotypes, are represented in (A), while RNAi lines with observable mild to lethal qualitative wing phenotypes are represented in (B). Boxplots indicate median (center line), 25th and 75th percentiles (bounds of box), and minimum and maximum (whiskers), with red dotted lines representing the control median. (PDF) [file pgen.1008792.s003.pdf]

**A** Larval *Drosophila* expression

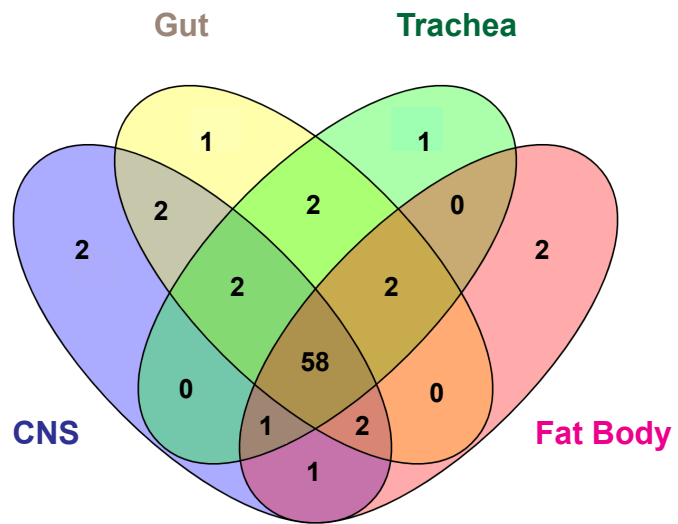

**B** Adult *Drosophila* expression

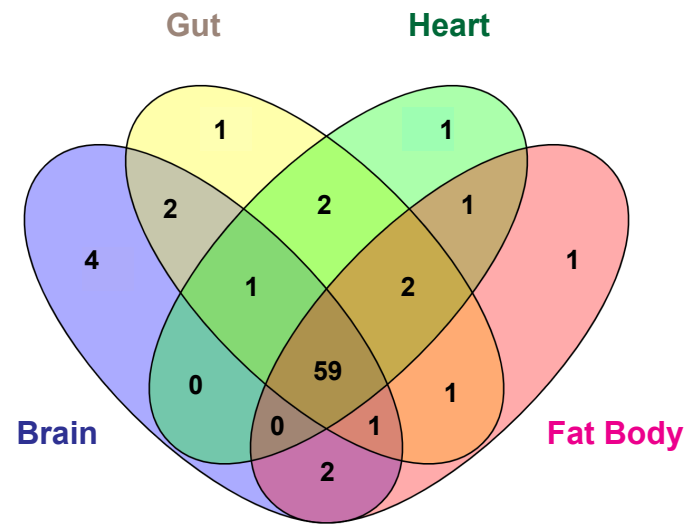

Supplement: S4 Fig — Venn diagrams representing the number of 76/77 fly homologs of CNV and neurodevelopmental genes that are expressed (>10 fragments per kilobase of transcript per million reads, or FPKM) in (A) larval (central nervous system or CNS, gut, trachea, and fat body) and (B) adult tissues (brain, gut, heart and fat body) are shown. (PDF) [file pgen.1008792.s004.pdf]

# Cellular processes in larval wing discs

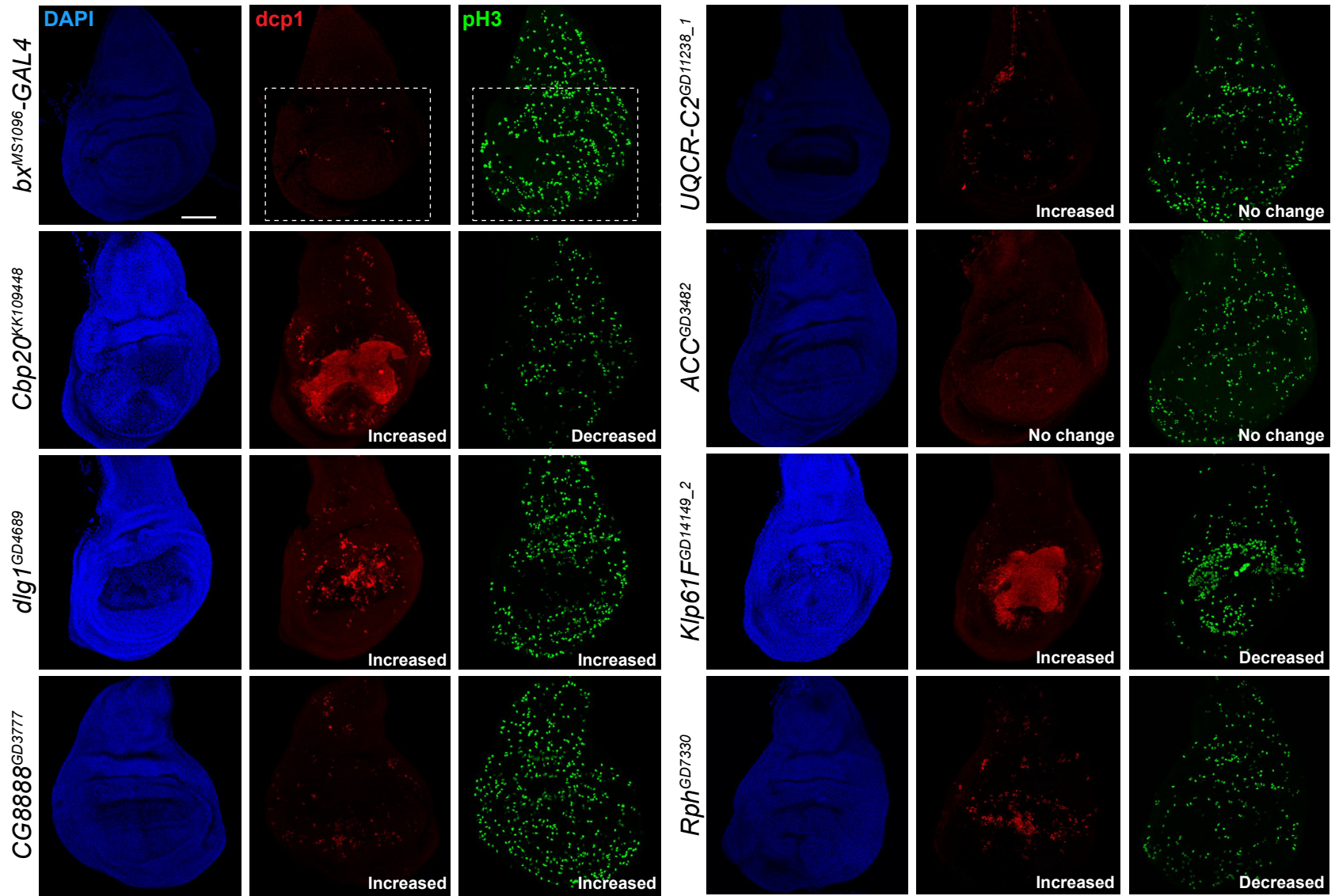

Supplement: S5 Fig — Larval imaginal wing discs (scale bar = 50 μm) stained with nuclear marker DAPI, apoptosis marker dcp1, and cell proliferation marker pH3 illustrate altered levels of apoptosis and cell proliferation due to wing-specific knockdown of select fly homologs of CNV and neurodevelopmental genes. We examined changes in the number of stained cells within the wing pouch of the wing disc (white box), which becomes the adult wing. Genotypes for the wing images are: w1118/bxMS1096-GAL4;+; UAS-Dicer2/+, w1118/bxMS1096-GAL4;UAS-Cbp20KK109448 RNAi/+; UAS-Dicer2/+, w1118/bxMS1096-GAL4;+; UAS-dlg1GD4689 RNAi/UAS-Dicer2, w1118/bxMS1096-GAL4;UAS-CG8888GD3777 RNAi/+; UAS-Dicer2/+, w1118/bxMS1096-GAL4;+; UAS-UQCR-C2GD11238 RNAi/UAS-Dicer2, w1118/bxMS1096-GAL4;+; UAS-ACCGD3482 RNAi/UAS-Dicer2, w1118/bxMS1096-GAL4;UAS-Klp61FGD14149 RNAi/+; UAS-Dicer2/+, and w1118/bxMS1096-GAL4;UAS-RphGD7330 RNAi/+;UAS-Dicer2/+. (PDF) [file pgen.1008792.s005.pdf]

# Disruption of signaling pathways in larval wing discs

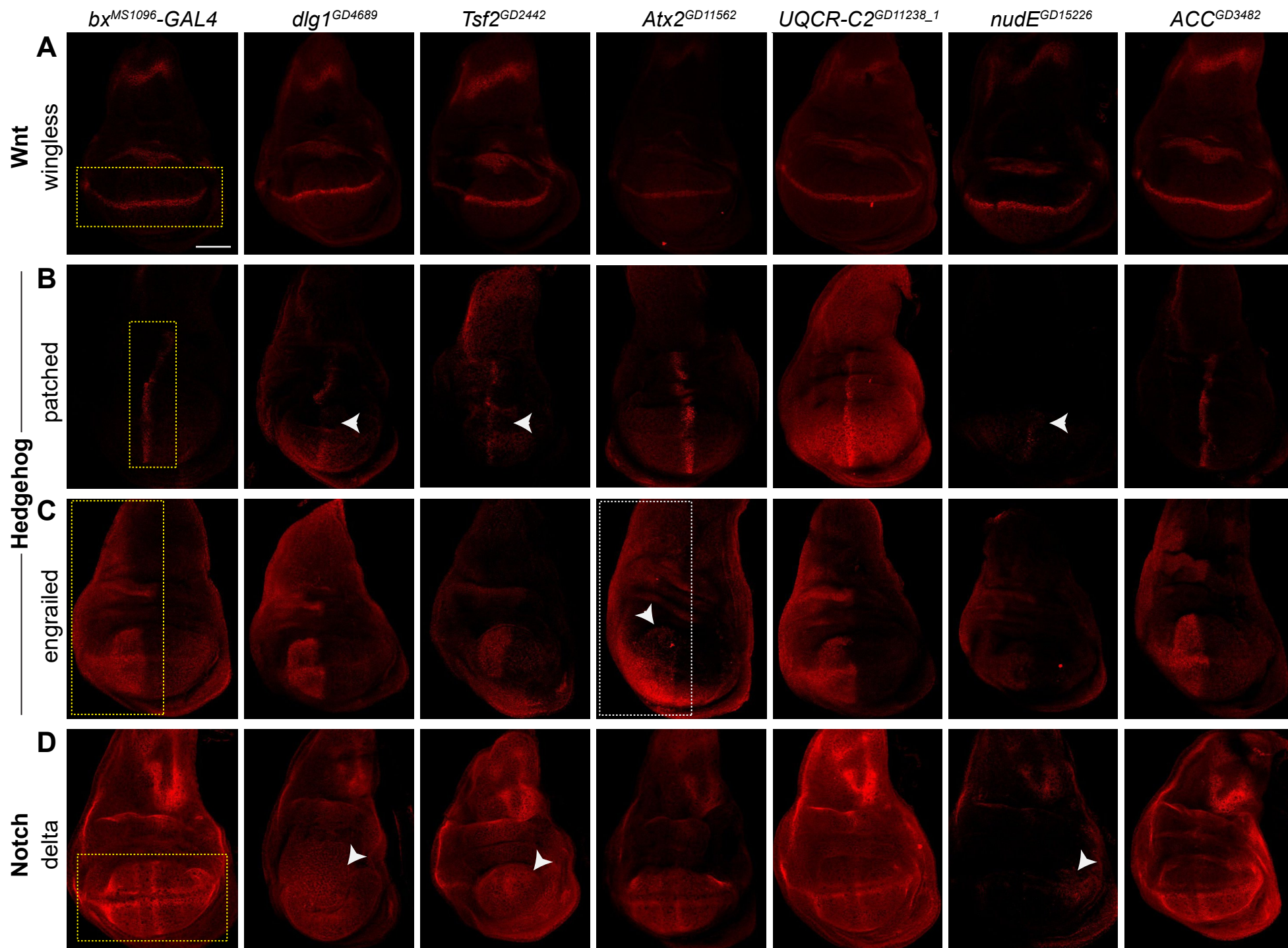

Supplement: S7 Fig — Larval imaginal wing discs (scale bar = 50 μm) stained with (A) wingless, (B) patched, (C) engrailed, and (D) delta illustrate disrupted expression patterns for proteins located within the Wnt (wingless), Hedgehog (patched and engrailed), and Notch (delta) signaling pathways due to wing-specific knockdown of additional fly homologs of CNV and neurodevelopmental genes. Dotted yellow boxes represent expression patterns for signaling proteins in bxMS1096-GAL4 control images. White arrowheads and dotted white boxes highlight disruptions in expression patterns of signaling proteins with knockdown of CNV genes. Genotypes for the wing images are: w1118/bxMS1096-GAL4;+; UAS-Dicer2/+, w1118/bxMS1096-GAL4;+; UAS-dlg1GD4689 RNAi/UAS-Dicer2, w1118/bxMS1096-GAL4;+; UAS-Tsf2GD2442 RNAi/UAS-Dicer2, w1118/bxMS1096-GAL4;+; UAS-Atx2GD11562 RNAi/UAS-Dicer2, w1118/bxMS1096-GAL4;+; UAS-UQCR-C2GD11238 RNAi/UAS-Dicer2, w1118/bxMS1096-GAL4;+; UAS-nudEGD15226 RNAi/UAS-Dicer2, and w1118/bxMS1096-GAL4;+; UAS-ACCGD3482 RNAi/UAS-Dicer2. (PDF) [file pgen.1008792.s007.pdf]
