# Supplementary material for: Drosophila models of pathogenic copy-number variant genes show global and non-neuronal defects during development
Source: PLoS Genet. 2020 Jun 24;16(6):e1008792. doi: 10.1371/journal.pgen.1008792 (PMC7313740; doi:10.1371/journal.pgen.1008792)
Supplement: S2 Fig — Boxplots show (A) L2, (B) L4, and (C) L5, longitudinal veins, and (D) anterior crossvein (ACV) and (E) posterior crossvein (PCV) lengths, for knockdown of select homologs in adult fly wings (n = 9–91, *p < 0.05, two-tailed Mann–Whitney test with Benjamini-Hochberg correction). Boxplots indicate median (center line), 25th and 75th percentiles (bounds of box), and minimum and maximum (whiskers), with red dotted lines representing the control median. (PDF) [file pgen.1008792.s002.pdf]

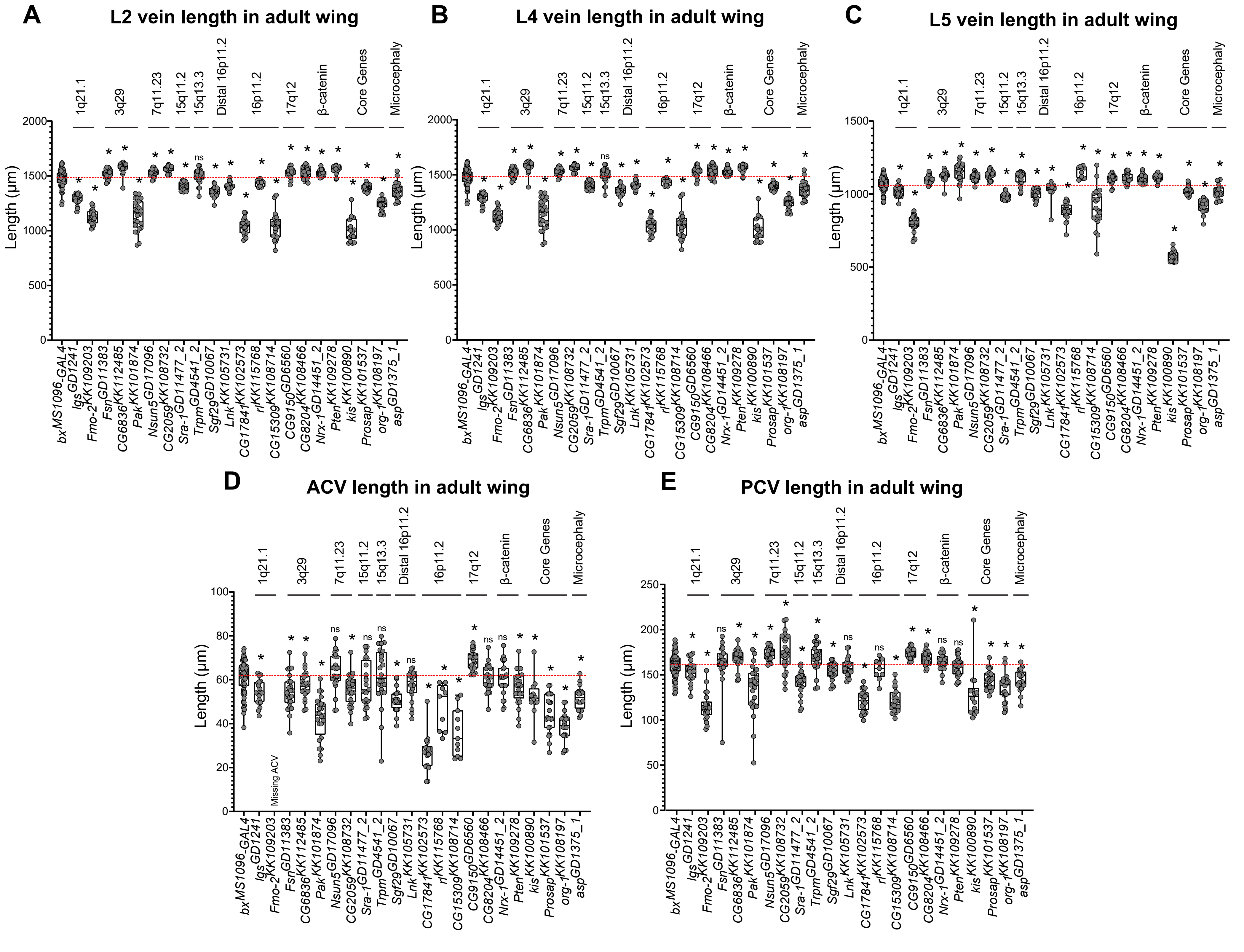

A
L2 vein length in adult wing
B
L4 vein length in adult wing
C
L5 vein length in adult wing
D
ACV length in adult wing
E
PCV length in adult wing
Length (µm)
1q21.1
3q29
7q11.23
15q11.2
15q13.3
Distal 16p11.2
16p11.2
17q12
β-catenin
Core Genes
Microcephaly
Missing ACV
ns
bx^MS1096-GAL4
lgs^GD1241
Fmo-2^KK109203
Fsn^GD11383
CG6836^KK112485
Pak^KK101874
Nsun5^GD17096
CG2059^KK108732
Sra-1^GD11477_2
Trpm^GD4541_2
Sgf29^GD10067
Lnk^KK105731
CG17841^KK102573
rl^KK115768
CG15309^KK108714
CG9150^GD6560
CG8204^KK108466
Nrx-1^GD14451_2
Pten^KK109278
kis^KK100890
Prosap^KK101537
org-1^KK108197
asp^GD1375_1
