# Supplementary material for: Drosophila models of pathogenic copy-number variant genes show global and non-neuronal defects during development
Source: PLoS Genet. 2020 Jun 24;16(6):e1008792. doi: 10.1371/journal.pgen.1008792 (PMC7313740; doi:10.1371/journal.pgen.1008792)
Supplement: S6 Fig — (A) Larval imaginal wing discs (scale bar = 50 μm) stained with nuclear marker DAPI, apoptosis marker dcp1, and cell proliferation marker pH3 illustrate altered levels of apoptosis and cell proliferation due to wing-specific knockdown of select fly homologs of CNV genes in females and males. We examined changes in the number of stained cells within the wing pouch of the wing disc (white box), which becomes the adult wing. Genotypes for the wing images are: w1118/bxMS1096-GAL4;+; UAS-Dicer2/+, w1118/bxMS1096-GAL4;+; UAS-lgsGD1241 RNAi/UAS-Dicer2, w1118/bxMS1096-GAL4;+; UAS-Sra-1GD11477 RNAi/UAS-Dicer2, and w1118/bxMS1096-GAL4;UAS-CG11035KK101201 RNAi/+; UAS-Dicer2/+. (B) Boxplot shows dcp1-positive cells in larval wing discs with knockdown of select fly homologs of CNV and neurodevelopmental genes, normalized to controls (n = 9–13, *p < 0.05, two-tailed Mann–Whitney test with Benjamini-Hochberg correction). (C) Boxplot shows pH3-positive cells in the larval wing discs with knockdown of select fly homologs of CNV and neurodevelopmental genes, normalized to controls (n = 9–13, *p < 0.05, two-tailed Mann–Whitney test with Benjamini-Hochberg correction). Boxplots indicate median (center line), 25th and 75th percentiles (bounds of box), and minimum and maximum (whiskers), with red dotted lines representing the control median. (PDF) [file pgen.1008792.s006.pdf]

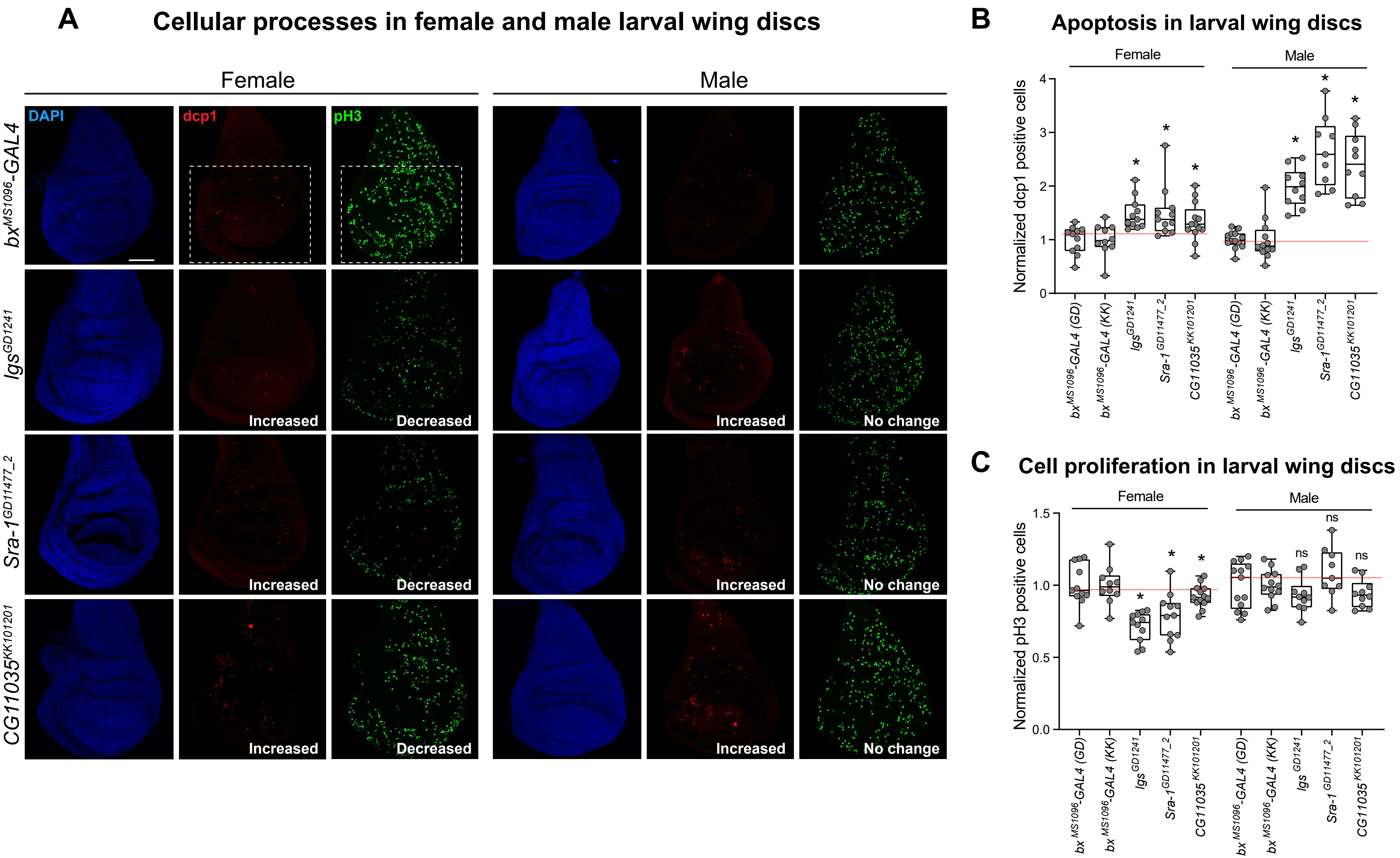

A
Cellular processes in female and male larval wing discs
Female
Male
DAPI
dcp1
pH3
bxMS1096-GAL4
lgsGD1241
Sra-1GD11477_2
CG11035KK101201
Increased
Decreased
No change
B
Apoptosis in larval wing discs
Female
Male
Normalized dcp1 positive cells
0
1
2
3
4
*
bxMS1096-GAL4 (GD)
bxMS1096-GAL4 (KK)
lgsGD1241
Sra-1GD11477_2
CG11035KK101201
C
Cell proliferation in larval wing discs
Normalized pH3 positive cells
0.0
0.5
1.0
1.5
ns
