# Supplementary material for: Drosophila models of pathogenic copy-number variant genes show global and non-neuronal defects during development
Source: PLoS Genet. 2020 Jun 24;16(6):e1008792. doi: 10.1371/journal.pgen.1008792 (PMC7313740; doi:10.1371/journal.pgen.1008792)
Supplement: S8 Fig — Network diagrams for connectivity between nine human CNV and neurodevelopmental genes whose fly homologs disrupt the (A) Wnt and (B) Hedgehog signaling pathways and 162 human Wnt and 46 human Hedgehog signaling genes within kidney, heart, and brain-specific gene interaction networks are shown. Yellow nodes represent CNV and neurodevelopmental genes, pink nodes represent Wnt or Hedgehog signaling pathway genes, and green nodes represent connector genes within the shortest paths between CNV and signaling pathway genes. (PDF) [file pgen.1008792.s008.pdf]

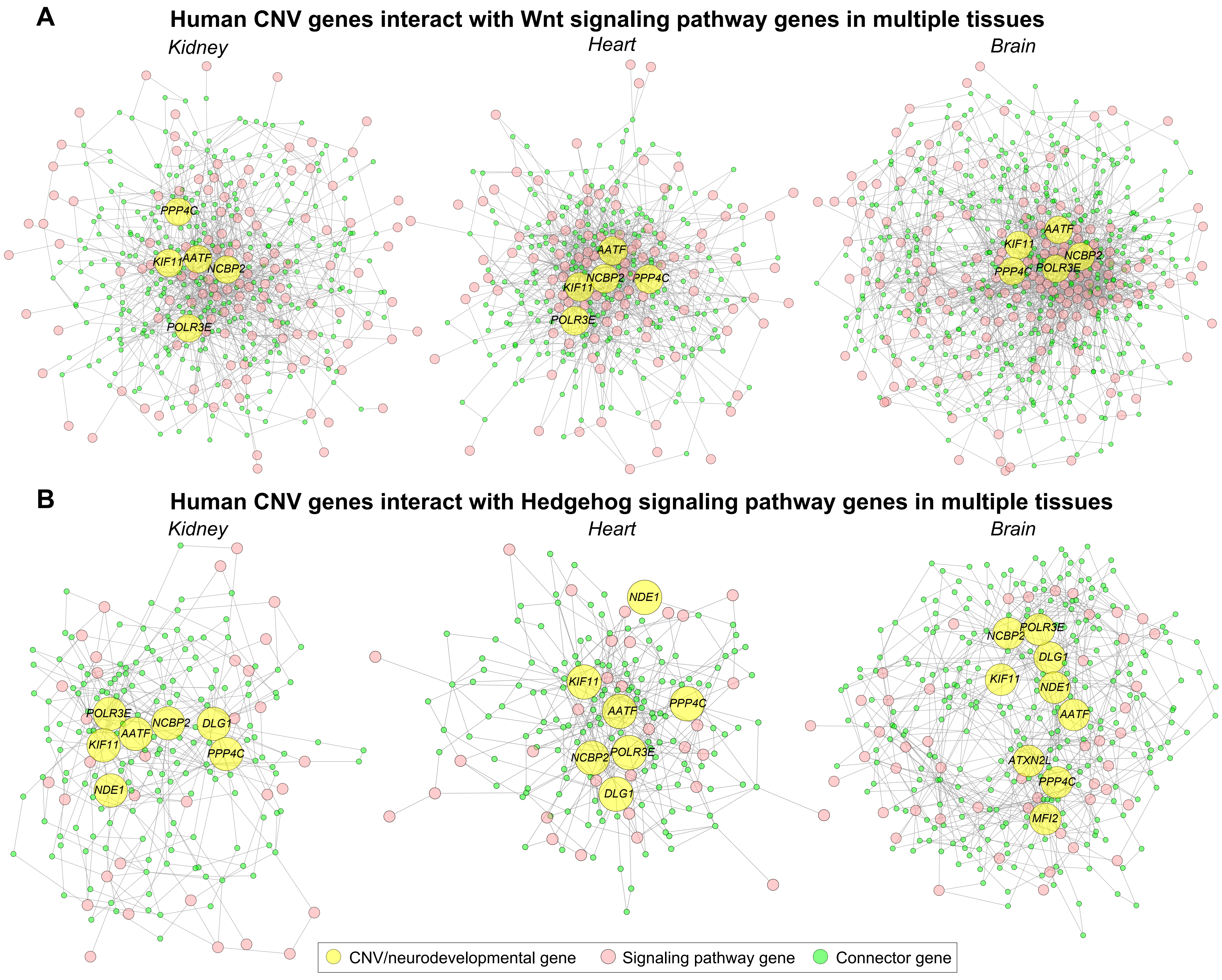
A
Human CNV genes interact with Wnt signaling pathway genes in multiple tissues
Kidney
PPP4C
KIF11
AATF
NCBP2
POLR3E
Heart
AATF
NCBP2
PPP4C
KIF11
POLR3E
Brain
AATF
KIF11
NCBP2
PPP4C
POLR3E
B
Human CNV genes interact with Hedgehog signaling pathway genes in multiple tissues
Kidney
POLR3E
AATF
NCBP2
DLG1
KIF11
PPP4C
NDE1
Heart
NDE1
KIF11
AATF
PPP4C
NCBP2
POLR3E
DLG1
Brain
NCBP2
POLR3E
DLG1
KIF11
NDE1
AATF
ATXN2L
PPP4C
MFI2
CNV/neurodevelopmental gene
Signaling pathway gene
Connector gene
