# Supplementary material for: Drosophila models of pathogenic copy-number variant genes show global and non-neuronal defects during development
Source: PLoS Genet. 2020 Jun 24;16(6):e1008792. doi: 10.1371/journal.pgen.1008792 (PMC7313740; doi:10.1371/journal.pgen.1008792)
Supplement: Attachment — Submitted filename: Yusuff et al reviewer response.pdf [file pgen.1008792.s020.pdf]

POINT-BY-POINT RESPONSES TO REVIEWER COMMENTS

RESPONSES TO REVIEWER #1 COMMENTS

1. ***While the authors provide intriguing data that identifies some of the likely contributors to the non-neuronal phenotypes, it would strengthen the paper if the authors could include in the study knockdown of a random selection of genes from a genomic interval of similar size to the pathogenic CNVs considered. For example, if 10/12 genes of a pathogenic CNV show non-neuronal phenotypes of varying severity, would a random selection of 10/12 genes from a similarly sized different region of the genome not associated with a pathogenic CNV give a similar result, or would the number of genes associated with phenotypes be reduced in number or severity? If the latter were the case, this would provide a strong argument that the pathogenic CNVs considered in this study are indeed enriched for developmentally important genes, and that flies are a reasonable proxy for identifying them.***

Response: We thank the reviewer for this important comment. We tested whether the fly homologs of genes within pathogenic CNVs are more likely to show developmental phenotypes than fly homologs of genes drawn from random human genomic intervals simulating different sized CNVs. We first generated 500-kbp (small CNV) and 1.5-Mbp (large CNV) genomic intervals from the start coordinate of each hg19 RefSeq gene in the human genome (n=18,625 total genomic intervals for both small and large CNVs), and identified fly homologs of genes within each region using the DIOPT v.7.1 homology tool (Hu et al., 2011). We then assessed the number of fly homologs of genes within each interval, normalized to the total number of homologs within that interval, that showed lethality with ubiquitous (*Act5C-GAL4*) knockdown, derived from a screen performed by Dietzl and colleagues (Dietzl et al., 2007); and wing phenotypes within mutant, RNAi, or overexpression lines derived from FlyBase (Thurmond et al., 2019) (**Fig. R1**). We found that the 3q29 region showed an enrichment for fly homologs with lethal phenotypes compared with fly homologs from simulated sets of 1.5-Mbp sized genomic intervals (6/14 genes, p=0.048), while the 17q12 region (3/11 genes, p=0.175) showed a non-significant enrichment of lethal genes (**Fig. R1A-B**). Furthermore, the distal 16p11.2 (4/6 genes, p=0.072) and 15q11.2 (2/4 genes, p=0.186) regions showed non-significant increases for fly homologs which showed wing phenotypes compared with fly homologs from simulated sets of 500-kbp genomic intervals (**Fig. R1C-D**).

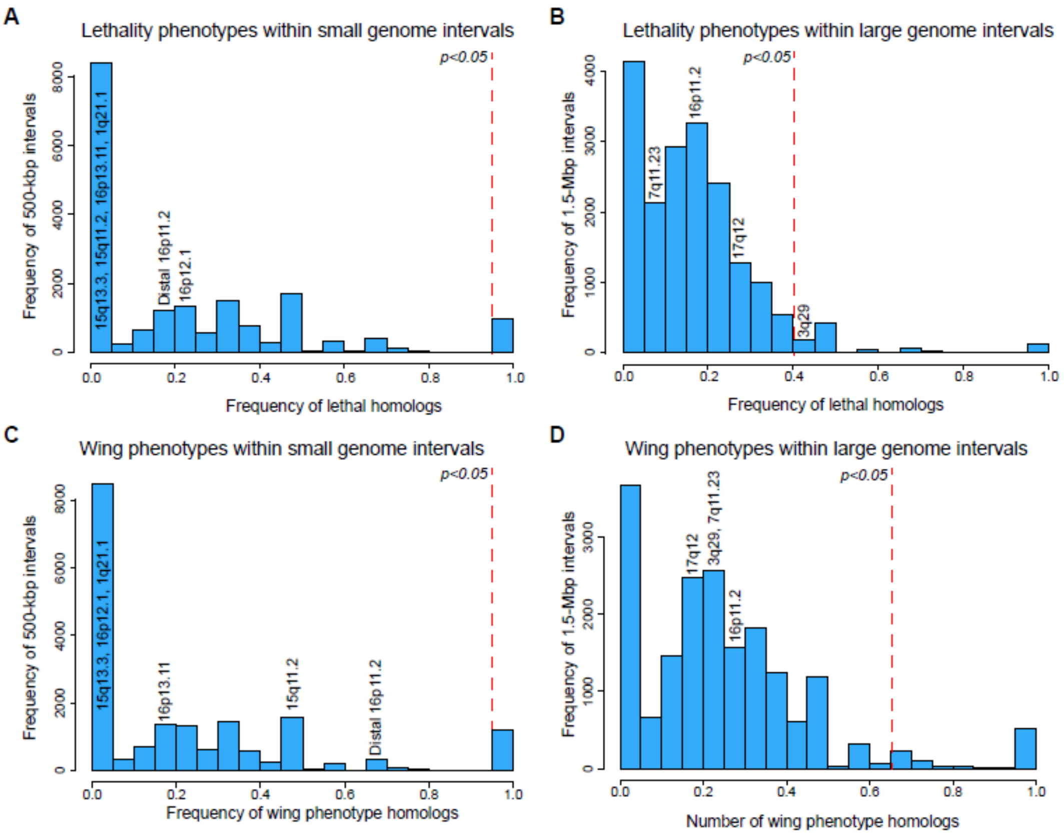

Figure R1. Frequency of fly homologs with lethal (top) or wing phenotypes (bottom) for human genes within simulated small (left, 500-kbp intervals) and large (right, 1.5-Mbp intervals) genomic intervals across the human genome (n=18.625 intervals for both small and large CNVs). Each plot highlights the frequency of fly homologs with lethal (**A-B**) or wing (**C-D**) phenotypes within the genomic intervals for ten pathogenic CNV regions tested in this study. Dotted red lines indicate 95th percentile, corresponding to an empirical $p<0.05$.

Overall, we found that most CNV regions were not enriched for fly homologs exhibiting developmental phenotypes. <u>This suggests that knockdown of random sets of genes outside of pathogenic CNV regions would potentially lead to a similar occurrence and severity of developmental phenotypes as those observed in our study.</u> The following points are noteworthy:

A. Our observations of lethality or wing abnormalities for fly homologs of both CNV and non-CNV genes indicate that a large number of genes in the fly genome contribute towards developmental phenotypes. In fact, Dietzl and colleagues found that an estimated 31.9% of fly genes are "essential" genes that lead to lethality with ubiquitous knockdown (Dietzl et al., 2007). Furthermore, our analysis of phenotypic data curated from FlyBase found that approximately 25.0% of fly genes with human homologs contribute towards wing abnormalities. Thus, we would expect that a random subset of fly homologs of human genes both within and outside of CNV regions would contribute towards these observed phenotypes.

B. Similarly, the lack of enrichment of fly homologs that exhibit lethal or wing phenotypes within pathogenic CNV regions could be due to the large number of genes both within and

outside of pathogenic CNVs that contribute towards developmental disorders in humans. To test this, we examined probability of loss-of-function intolerance (pLI) scores (Lek et al., 2016), a proxy for gene pathogenicity, of human genes within the 10 rare CNVs that we tested in this study. We found no significant differences (p=0.116, two-tailed Mann-Whitney test) in pLI scores among CNV genes (average pLI=0.346, n=130) compared with all other genes in the human genome (average pLI=0.3061, n=18,496). This highlights that genes both within and outside of pathogenic CNVs are equally likely to contribute towards disease pathogenicity. These results also correspond to previous findings by Ji and colleagues, who identified approximately 4,000 genes across the mammalian genome that are essential for development (Ji et al., 2016). Furthermore, numerous genes both within and outside of CNV regions have been associated with syndromic forms of developmental phenotypes, such as cardiac defects (Richards and Garg, 2010) and craniofacial defects (Twigg and Wilkie, 2015).

C. The enrichment of pathogenic CNVs among individuals with developmental disorders is primarily due to the underlying genomic architecture, where flanking segmental duplications predispose these regions to deletion or duplication events (Girirajan and Eichler, 2010). In fact, there is an approximately 25-fold enrichment of CNVs within genomic hotspots flanked by segmental duplications compared to the rest of the genome (Itsara et al., 2008). While we focused on these recurrent pathogenic CNVs in this manuscript, many other regions of the genome also harbor pathogenic CNVs that may not be recurrent due to the lack of flanking segmental duplications. For example, 77.0% of the human genome contains CNVs between 50 kbp-10 Mbp in size that were categorized as pathogenic within the ClinGen database (Kaminsky et al., 2011), indicating that dosage-sensitive genes which contribute to disease are pervasive throughout the genome. Thus, the lack of enrichment of fly homologs exhibiting developmental phenotypes within pathogenic CNV regions is a reflection of the random composition of genes within these regions.

Moreover, our results from *Drosophila* studies are useful in identifying key CNV genes that contribute towards developmental phenotypes. Although assessment of genes outside of CNV regions would likely uncover similar individual candidate genes, we used *Drosophila* as a model system to assess homologs of genes within 10 pathogenic CNVs towards conserved non-neuronal features and underlying cellular pathways (Bellen et al., 2010; Bier, 2005; Wangler et al., 2015). Our study highlights the roles of several candidate homologs of genes within CNV regions (such as *POLR3E* within 16p12.1, *NCBP2* within 3q29, and *PPP4C* within 16p11.2) towards specific cellular processes and signaling pathways (such as proliferation, apoptosis, and Wnt pathway) relevant to the observed developmental phenotypes. We have included these points in the Introduction of our manuscript:

> ***Introduction, page 5-6:*** *"Drosophila melanogaster is an excellent model system to evaluate homologs of neurodevelopmental genes, as many developmental processes and signaling pathways are conserved between humans and flies [27]. ... Although fly wing phenotypes cannot be directly translated to human phenotypes, defects observed in fly wings can be used to assess how homologs of human disease genes alter conserved cellular and developmental mechanisms. ... Hence, the fly wing provides a model system that is ideal for evaluating the contributions of individual homologs of CNV genes towards cellular and developmental defects."*

2. ***It would help to have a figure that lays out how many genes per CNV would need to be considered for knockdown (the total number), and how many of those have fly orthologs that could be tested (Table S1 could be expanded for this). In addition, any genes within the CNVs known to be involved in any of the phenotypic anomalies present in cases carrying that CNV should be highlighted. Collectively, this would help to determine whether known genes responsible for at least a subset of the phenotypes (and are thus critically important) are able to be tested in flies. Finally, along the lines of the lgs/Fmo-2 and asp, it would be nice to show that additional phenotypes of the knockdowns of such fly genes have some relationship to the phenotypic anomalies in cases (e.g., a gene knockdown in flies resulting in reduced proliferation could be connected to a hypoplastic organ in cases).***

Response: To clarify the reviewer's comment, we note that we tested 59 total fly homologs of human CNV genes out of 130 total genes in these CNV regions (45.4%). We did not test fly models if the human CNV genes did not have a fly homolog, the fly homolog did not have an available RNAi line, or the available RNAi lines did not pass quality control. We have revised the Results and Methods to clarify this point in our manuscript:

> ***Results, page 8:*** *"We used the bxMS1096-GAL4 wing-specific driver to assess a total of 136 RNAi lines for 59 fly homologs of 130 total human genes within pathogenic CNV regions (chromosomal locations 1q21.1, 3q29, 7q11.23, 15q11.2, 15q13.3, 16p11.2, distal 16p11.2, 16p12.1, 16p13.11, and 17q12), as well as 20 fly homologs of human genes associated with neurodevelopmental disorders (S2 Data)."*

> ***Methods, Page 23:*** *"We selected Drosophila homologs for 59 human genes out of 130 total genes located within 10 pathogenic CNV regions [89] associated with neurodevelopmental disorders (1q21.1, 3q29, 7q11.23, 15q11.2, 15q13.3, 16p11.2, distal 16p11.2, 16p12.1, 16p13.11, and 17q12) (S2 Data). These 59 genes were selected based on presence of fly homologs, availability of RNAi lines for fly homologs, and quality control of the RNAi lines."*

We have further expanded **S2 Data** (previously **S1 Data**) to include 71 human CNV genes and available fly homologs and DIOPT scores that we did not test in this manuscript, along with the 59 CNV genes that were tested in this study (see excerpt below as **Table R1**).

Table R1. *Drosophila* homologs of human CNV and neurodevelopmental genes

CNV region	Human gene	Fly homolog	DIOPT score	Weighted score	% identity	Rank
Distal 16p11.2	*ATP2A1*	*SERCA*	14	13.82	71.82%	high
Distal 16p11.2	*ATXN2L*	*Atx2*	11	10.99	24.54%	high
Distal 16p11.2	*CCDC101*	*Sgf29*	14	13.8	50.00%	high
Distal 16p11.2	*CD19*	*GMF*	1	1.01	38.46%	low
Distal 16p11.2	*LAT*	None	0	0		
Distal 16p11.2	*NFATC2IP*	*Rad60*	2	1.95	19.15%	high
Distal 16p11.2	*RABEP2*	*Rbpn-5*	3	2.91	23.63%	moderate

Distal 16p11.2	*SH2B1*	*Lnk*	9	8.75	28.39%	moderate
Distal 16p11.2	*SPNS1*	*spin*	13	12.79	48.28%	high
Distal 16p11.2	*TUFM*	*efTuM*	13	12.86	62.22%	high
16p12.1	*CDR2*	*Cen*	5	4.89	22.68%	moderate
16p12.1	*EEF2K*	None	0	0		
16p12.1	*MOSMO*	*CG14182*	7	6.92	51.50%	high
16p12.1	*PDZD9*	None	0	0		
16p12.1	*POLR3E*	*Sin*	10	9.77	32.42%	high
16p12.1	*UQCRC2*	*UQCR-C2*	15	14.75	33.49%	high
16p12.1	*VWA3A*	None	0	0		

Drosophila homologs of human CNV and neurodevelopmental genes, as determined using DIOPT v.7.1. Grey shading indicates human CNV genes that were not assessed in this study.

We also note that a subset of CNV genes have been associated with specific developmental phenotypes, such as *ELN* for supravalvular aortic stenosis within the 7q11.23 deletion (Ewart et al., 1993), and *GJA8* for cataracts and *GJA5* for heart defects within the 1q21.1 deletion (Ponnam et al., 2007; Soemedi et al., 2012). However, we were not able to test these specific genes, as none of them have fly homologs. The main purpose of the manuscript is to understand how homologs of CNV genes contribute to cellular mechanisms that could underlie developmental defects associated with pathogenic CNVs. While the cellular phenotypes we tested in fly models are conserved in humans, future follow-up studies would be necessary to directly correlate these cellular phenotypes with specific developmental defects. For example, further analysis of cardiac defects due to knockdown of CNV homologs in *Drosophila*, as illustrated in (Grossman et al., 2011), would provide deeper mechanistic insights. More broadly, follow-up studies in mammalian systems could confirm whether decreased proliferation that we observed with knockdown of fly homologs of CNV genes could be responsible for congenital defects observed in CNV carriers. We have included these points in the Discussion of our manuscript:

> ***Discussion, page 22***: *"Our study further exemplifies the utility of evaluating non-neuronal phenotypes in addition to neuronal phenotypes in functional models of individual genes and CNV regions associated with developmental disorders, including future studies in mammalian or cellular model systems. Further studies exploring how CNV genes interact with each other and with other developmental pathways could more fully explain the conserved mechanisms underlying global developmental defects and identify potential therapeutic targets for these disorders."*

3. ***It is unclear why wing-specific drivers would result in lethality unless the driver is not specific for wing expression. A second driver could be used for at least a subset of genes to address this issue, and confirm/refute the initial results. If a truly wing-specific driver were used, then knockdown of these genes should reveal a phenotype (since wing development is not required for viability).***

Response: We thank the reviewer for bringing up this important point. The wing-specific *bx*^{MS1096}-*GAL4* driver, which was used in the manuscript, is primarily expressed in the imaginal

wing discs in the larval and pre-pupal stages (Capdevila and Guerrero, 1994; Neumann and Cohen, 1996), with some reports of expression in embryonic salivary glands (Marquez et al., 2001). While the wing is not required for viability, the lethality observed with wing-specific knockdown of CNV genes highlights the essentiality of these genes during early developmental stages. In fact, several studies have identified lethal phenotypes with misexpression of fly genes using wing-specific drivers such as *bx*^{MS1096}, *en,* or *71B-GAL4* (Bischof et al., 2012; Jacobsen et al., 2006; Morris et al., 2006; Straub et al., 2020). Furthermore, similar lethality has been observed with other tissue-specific *GAL4* drivers, such as eye-specific *GMR-GAL4* and *DE-GAL4* (Iyer et al., 2018; Morris et al., 2006; Singh et al., 2020; Zhu et al., 2017), despite the fly eye being dispensable for genetic perturbations (Kumar, 2012).

4. ***How well does DIOPT predict orthologues? For example, many of the genes tested are ranked as moderate, with a range of DIOPT scores. This should be explained, including how much actual conservation exists for "moderate" genes with the various DIOPT scores.***

Response: The *DRSC* Integrative Ortholog Prediction Tool (DIOPT) has been extensively used by *Drosophila* researchers to identify fly homologs of human genes (Shulman et al., 2014; Vinayagam et al., 2016; Wangler et al., 2017). DIOPT provides both scores and ranks (i.e. low, moderate, high) for pairs of homologs in model organisms (Hu et al., 2011). The ranks for each homolog pair are based on the numeric scores, determined by the number of orthology prediction tools queried by DIOPT that indicate orthology between the two genes, and whether the two genes are the best matches for each other in their respective genomes. For example, "high" rankings are determined if the score is >2 and each gene in the pair is the best match for each other, while "moderate" rankings are determined if the score is >2 and one of the genes is the best match for the other gene. We identified fly homologs of human genes based on the highest numeric score, and excluded genes with "low" rankings (score <2).

In addition to scores and rankings, DIOPT also provides the % amino acid identity between pairs of homologs, which show a significant correlation with the numeric DIOPT scores (Pearson's correlation, r=0.5488, p<0.0001) (**Fig. R2**). Based on the reviewer's comment, we have now added the DIOPT % identity to **S2 Data** (except shown above as **Table R1**). We have also clarified the use of DIOPT to identify fly homologs of human genes in the revised Methods:

> ***Methods, page 23***: *"We used the DRSC Integrative Ortholog Prediction Tool (DIOPT, v.7.1) [42] to select fly homologs for each human gene based on highest numeric score, excluding genes with "low" rankings (score <2) (S2 Data)."*

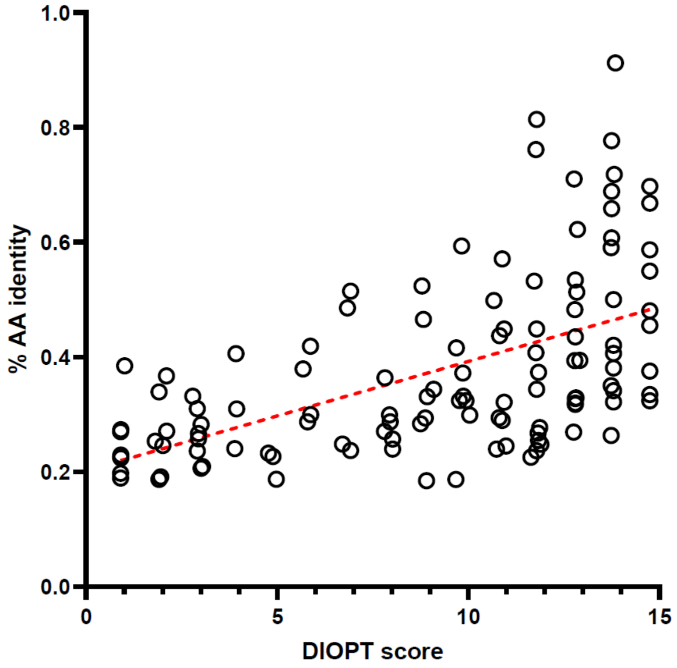

Figure R2. Correlation between DIOPT scores and % amino acid identity for pairs of human genes and fly homologs tested in this study.

5. ***The eye-specific knockdown experiments are meant to look at "neuronal defects," but it is never explained why knockdown in the eye should give rise to neuronal defects. This needs to be explained.***

Response: The *Drosophila* eye has been classically used to model human neurological disorders, such as Alzheimer's disease (Wittmann et al., 2001), Rett syndrome (Cukier et al., 2008), Spinocerebellar ataxia type 3 (Bilen and Bonini, 2007), and Huntington's disease (Jackson et al., 1998). In fact, we recently used the fly eye to identify homologs of genes within the neurodevelopmental-associated 16p11.2 and 3q29 deletion regions that contribute towards neuronal phenotypes, many of which were validated using pan-neuronal *Elav*-GAL4 mediated knockdown (Iyer et al., 2018; Singh et al., 2020). The *Drosophila* eye is comprised of individual ommatidial units that contain photoreceptor neurons, which project into the optic lobe within the brain, as well as non-neuronal pigment and accessory cells (Kumar, 2012; Nériec and Desplan, 2016). We have revised the Results to include details explaining the utility of *Drosophila* eye models towards understanding neuronal defects:

> ***Results, page 11-12**: "The Drosophila eye has been classically used to evaluate neuronal phenotypes due to genetic perturbations, as individual ommatidial units within the eye contain photoreceptor neurons that project into the optic lobe within the brain [49,50]. In fact, numerous genes associated with human neurological disorders have been modeled using the fly eye [51–53]."*

6. ***Since flies do not have structurally conserved major vertebrate organs such as kidneys and lungs, it is more challenging to test pathways involved in their morphogenesis. (minor criticism)***

Response: We agree with the reviewer that *Drosophila* do not have conserved human organs such as kidneys or lungs, as observed in mammalian systems. However, the molecular mechanisms and pathways which may underlie the observed clinical features in humans are conserved in *Drosophila* model systems (Bellen et al., 2010; Bier, 2005; Wangler et al., 2015). While we cannot directly test the involvement of CNV genes towards morphogenesis of vertebrate organs using fly models, our study narrows down potential candidate genes, processes and pathways for future studies in vertebrate model organisms.

RESPONSES TO REVIEWER #2 COMMENTS

1. ***Some of the numbers, I am having a hard time to keep clear. For instance, line 145-146 "We observed four clusters of RNAi lines: 75 lines with no observable qualitative phenotypes (55.2%)." That would suggest that 61 lines had observable qualitative phenotypes. but then in line 163- "For example, discoloration (87 lines in males compared to 56 lines in females)". That suggest that you observed discoloration in 87 lines. Does clustering group the mildest qualitative phenotypes as "no observable phenotypes" bin? If so, this could be clearer.***

Response: We apologize to the reviewer for the confusion regarding the descriptions of qualitative fly phenotypes in the results. We would like to clarify that the k-means clustering analysis in lines 145-146 applied only to the tested female lines (i.e. 75 lines without observable phenotypes, and 61 lines with observable phenotypes). In line 163, the numbers for fly lines showing discoloration are not based on the k-means clustering analysis, as we did not perform clustering analysis for the tested male lines. The 87 male lines and 55 female lines (*corrected from 56 in the manuscript*) indicate all lines which show any discoloration phenotypes, excluding lines that show severe and lethal phenotypes (see raw data in **Data S3**). As the 55 female lines include 22 lines within the "no observable phenotype" cluster, the reviewer is correct that lines with the mildest qualitative phenotypes are clustered into the "no observable phenotype" category by the k-means classifier. We have made the following changes to the manuscript to clarify these points:

> ***Results, page 8***: *"We categorized adult female wing phenotypes based on their severity, and performed k-means clustering analysis to categorize each RNAi line by their overall phenotypic severity (Fig. 2A-B)."*
>
> ***Results, page 9***: *"Certain qualitative phenotypes were present at a higher frequency in males compared to females among all non-lethal and non-severe RNAi lines (independent of k-means clustering analysis). For example, discoloration (87 lines in males compared to 55 lines in females; p=1.315×10-4, two-tailed Fisher's exact test) and missing vein phenotypes (92 lines in males compared to 29 lines in females; p=2.848×10-16, two-tailed Fisher's exact test) were more commonly observed in males than females (S3 Data)."*

2. ***Line 159-161: "We note that 18/79 fly homologs showed discordant phenotypes between two or more RNAi lines for the same gene, which could be due to differences in expression of the RNAi construct among these lines." Can this be confirmed with qPCR to see if certain RNAi lines are less effective than others?***

Response: We thank the reviewer for this comment. We noted in the manuscript that differences in knockdown levels among different RNAi lines for the same gene could account for the observed discordant phenotypes. While we did not test all 43 lines for the 18 discordant genes using qPCR, we examined qPCR data for a subset of discordant lines from our previously published data (Iyer et al., 2016, 2018) using a pan-neuronal driver (*Elav-GAL4*). For example, two RNAi lines for *C16orf53/Pa1* showed discordant phenotypes that could be explained by

significantly different gene expression levels: *Pa1*^{GD6674} showed no phenotype with 63.49% average expression, while *Pa1*^{KK110317} showed mild phenotypes with 35.33% average expression ($p=1.95\times10^{-4}$, two-tailed Student's t-test). Similarly for *UBE3A/Ube3a* RNAi lines, *Ube3a*$^{GD450\_1}$ showed 65.89% average gene expression with large L3 vein length and wing area phenotypes, while *Ube3a*$^{GD450\_2}$ showed 52.06% average expression with small wing area ($p=5.44\times10^{-3}$, two-tailed Student's t-test). Thus, the differences observed in phenotypes could potentially be explained by differences in gene expression among discordant RNAi lines.

3. ***It is not clear how the final total of 59 genes were determined. Did you identify the genes that would most likely affect nonneuronal phenotypes? Were they the only ones that had homologs and RNAi lines for? If the former, this could imply that their results might be an over estimation of the proportion of CNV genes that affect development. The authors do a good job not overstepping their results, but I feel like this should be discussed or clarified.***

Response: We apologize for not making this clear in the manuscript. We selected 59 human CNV genes (out of a total 130 CNV genes) to test for developmental phenotypes in *Drosophila* models based on presence of a fly homolog, availability of RNAi lines for the fly homolog, and quality control of the RNAi lines. To identify fly homologs of human CNV genes, we used the *DRSC* Integrative Ortholog Prediction Tool (DIOPT) (Hu et al., 2011). We identified fly homologs of human genes based on the highest numeric score provided by DIOPT, and excluded genes with "low" rankings (score <2). As such, our approach to selecting CNV genes was unbiased towards their likelihood of contributing towards non-neuronal phenotypes. We have now clarified these points in the revised Methods:

> ***Methods, page 23****: "We selected Drosophila homologs for 59 human genes out of 130 total genes located within 10 pathogenic CNV regions [89] associated with neurodevelopmental disorders (1q21.1, 3q29, 7q11.23, 15q11.2, 15q13.3, 16p11.2, distal 16p11.2, 16p12.1, 16p13.11, and 17q12) (S2 Data). These 59 genes were selected based on presence of fly homologs, availability of RNAi lines for fly homologs, and quality control of the RNAi lines."*

4. ***You removed the KK lines with the offsite tio target. Was this confirmed using the PCR assay or the crossing 40DUAS? This could be clearer. And if you used the PCR assay, I suggest the gel be a supplementary figure.***

Response: We thank the reviewer for seeking this clarification. We would like to note that we did not screen for *tio* overexpression in the KK RNAi lines assessed in our study using crosses with the *40DUAS* line (Vissers et al., 2016). Instead, we screened KK RNAi lines for wing inflation defects, a non-specific phenotype caused by off-target effects of *tio* overexpression with the pan-neuronal driver *Elav-GAL4*, as described by Green and colleagues (Green et al., 2014). We found such defects in 6/51 KK RNAi lines with *Elav-GAL4* mediated knockdown. Previously, we also used qPCR to confirm overexpression of *tio* in a subset of these lines with *Elav-GAL4* knockdown (**Table R2**) (Iyer et al., 2018; Singh et al., 2020).

Table R2. ***tio*** **overexpression in KK RNAi lines of CNV homologs**

Human gene	RNAi line	*tio* expression (%)	St. dev.
C16orf53	*Pa1*^{KK110317}	235.06%	10.639
FAM57B	*CG17841*^{KK102573}	215.23%	7.341
YPEL3	*CG15309*^{KK108714}	255.67%	11.574
TCTEX1D2	*CG5359*^{KK107839}	177.09%	12.742

Our previous screen for qualitative wing phenotypes among fly homologs of the 16p11.2 deletion with wing-specific *bx*^{MS1096}*-GAL4* knockdown did not show any wing inflation defects among RNAi lines with *tio* off-target effects (Iyer et al., 2018). This is similar to our finding that KK lines with eye-specific *GMR-GAL4* knockdown did not show similar eye phenotypes as flies with *GMR-GAL4* mediated overexpression of *tio* (Iyer et al., 2018). We therefore included these three RNAi lines of 16p11.2 homologs in our study (*Pa1*^{KK110317}, *CG17841*^{KK102573}, and *CG15309*^{KK108714}), which showed mild phenotypes including wrinkled wing, missing vein, or discoloration (**Fig. R3**). Thus, *bx*^{MS1096}*-GAL4* mediated *tio* off-target effects in KK RNAi lines do not contribute to severe wing defects, as observed with *Elav-GAL4* mediated knockdown. We note that we did not assess wing phenotypes for the other three KK RNAi lines that exhibited wing inflation defects upon *Elav-GAL4* knockdown. As we did not use pan-neuronal *Elav-GAL4* for any experiments in this study, and KK RNAi lines with *bx*^{MS1096}*-GAL4* knockdown did not show wing inflation defects, we have therefore corrected the text and removed the statement regarding RNAi lines with *tio* offsite targets from the revised Methods.

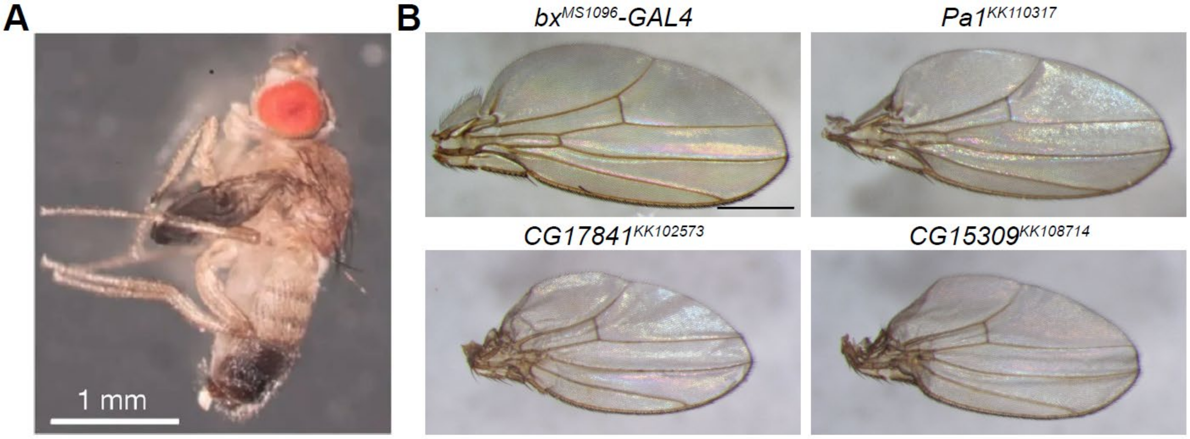

Figure R3. Comparison of wing phenotypes due to *tio* off-target effects in KK RNAi lines with *Elav-GAL4* and *bx*^{MS1096}*-GAL4* drivers. **A.** Adult fly displaying wing-inflation defects with *Elav-GAL4* mediated *tio* overexpression due to off-target effects of KK RNAi line (modified from (Green et al., 2014)). **B.** Representative brightfield images of adult wings (scale bar = 500µm) with *bx*^{MS1096}*-GAL4* knockdown of three KK RNAi lines with *tio* off-target effects show fully inflated wings with mild phenotypes.

5. ***Homologs had varied phenotypic effects. Looking at figure 2A, homologs associated with CNV 16p11.2 had a higher proportion of lethal and or severe phenotypes than the others.***

Can you compare the CNVs against each other? Does that CNV have more severe phenotypic impacts than the other CNVs. Is it possible to associate your finding with severity of the particular CNVs in humans?

Response: We agree with the reviewer that it would be interesting to correlate the severity of *Drosophila* phenotypes with the phenotypes observed in human CNV carriers. However, we could only make such conclusions after thoroughly comparing the effects of several CNV homologs in multiple tissues (i.e. eye, brain, wing, and ubiquitous knockdown) and assessing the nature of interactions between these homologs. We kindly note that these assessments would be beyond the scope of this study.

With regards to comparing the severity of CNVs in humans, previous results from our work have shown extensive variability of both neuronal and non-neuronal phenotypes for these pathogenic CNVs, which are also modulated by variants in the genetic background (Girirajan et al., 2012; Pizzo et al., 2019). In fact, we found that each of the 10 pathogenic CNVs we tested contribute to a range of variably-expressive phenotypes across multiple non-neuronal tissues among individuals within the DECIPHER database (Firth et al., 2009) (new **S1 Fig.**, shown below as **Fig. R4**; new **S1 Data**, excerpt shown below as **Table R3**).

We have now added our analysis of the DECIPHER database phenotypic data to the Introduction and Methods:

> ***Introduction, page 4:*** *"In fact, an overall survey of ten rare disease-associated CNVs among individuals within the DECIPHER database [9] showed a wide range of non-neuronal phenotypes across multiple organ systems for each CNV disorder (S1 Fig., S1 Data)."*

> ***Methods, page 29:*** *"Phenotypic data for human carriers of 10 pathogenic CNVs were obtained from the DECIPHER public database [9] (S1 Data). Clinical phenotypes for each CNV carrier were categorized by top-level Human Phenotype Ontology terms [100] using the Orange3 Bioinformatics software library (https://orange-bioinformatics.readthedocs.io), and the frequency of individuals carrying each top-level phenotype term was calculated for each CNV."*

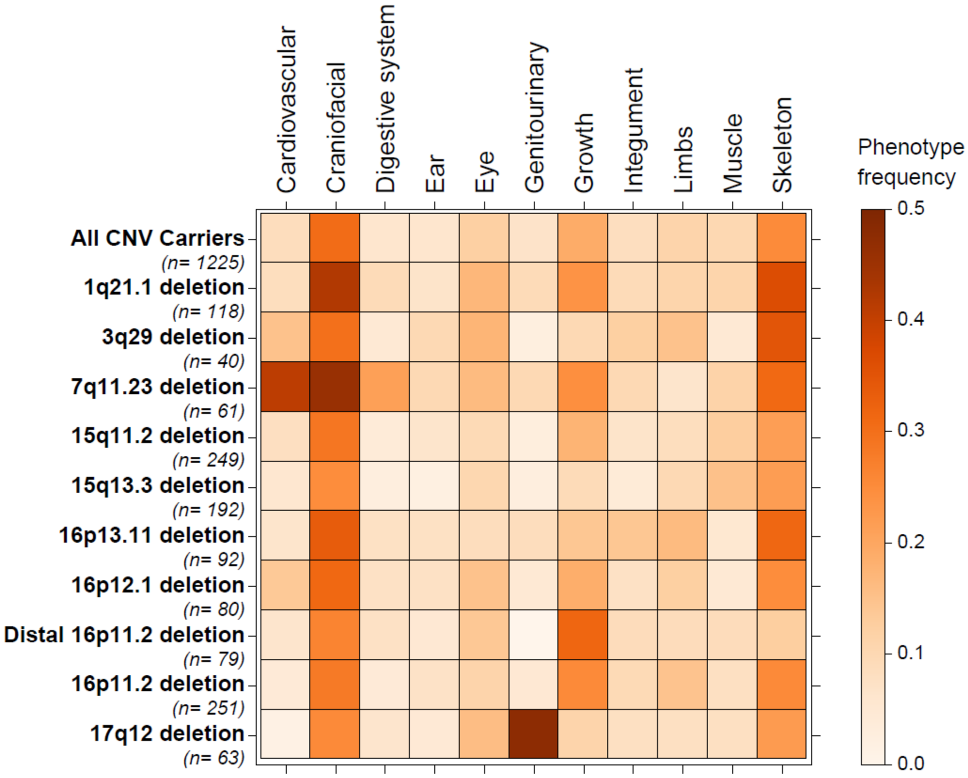

Figure R4. Heatmap with frequencies of non-neuronal developmental phenotypes observed in 1,225 human carriers of 10 pathogenic CNV deletions, curated from the DECIPHER database, is shown. CNV carriers show a variety of phenotypes that manifest across different tissues, including eye, limbs, muscle, and skeleton.

Table R3. Non-neuronal phenotypes of individuals carrying rare pathogenic CNVs in the DECIPHER database.

CNV region	Samples	Cardiovascular	Digestive	Head/ neck	Limbs	Musculature	Skeletal
15q11.2 deletion	249	20	10	72	21	32	54
		8.03%	4.02%	28.92%	8.43%	12.85%	21.69%
15q13.3 deletion	192	11	5	48	19	29	42
		5.73%	2.60%	25.00%	9.90%	15.10%	21.88%
16p11.2 deletion	251	11	11	71	37	20	64
		4.38%	4.38%	28.29%	14.74%	7.97%	25.50%
Distal 16p11.2 deletion	79	5	6	21	7	7	10
		6.33%	7.59%	26.58%	8.86%	8.86%	12.66%
16p12.1 deletion	80	11	6	25	10	4	20
		13.75%	7.50%	31.25%	12.50%	5.00%	25.00%
16p13.11 deletion	92	6	7	31	15	5	29
		6.52%	7.61%	33.70%	16.30%	5.43%	31.52%

Minor comments:

6. ***Some of the figure legends are long (like figure 1 and 4). In the case of figure 4, not much can be done but maybe for figure 1, part A could be its own figure.***

Response: We appreciate the reviewer's helpful comment, and have separated previous **Fig. 1A** into a new **Fig. 1**, with **Fig. 1B-D** now comprising **Fig. 2**. All subsequent Figures have also been relabeled, and minor text changes were made to reduce the length of **Fig. 5** legend (previously **Fig. 4**).

7. ***Figure 6B and 6C could be rearranged to show the pattern described in the text. Instead of having the X axis ordered by their effects with respect to apoptosis and proliferation. Order the genes by their wing severity. As done in figure 4B.***

Response: We thank the reviewer for their helpful suggestion, and have revised **Fig. 7B-C** (previously **Fig. 6B-C**, shown below as **Fig. R5**) to re-order the genes according to their observed wing phenotypic severity.

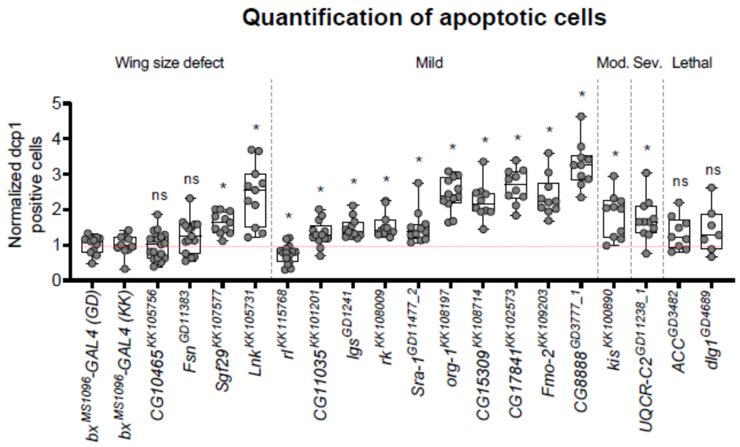

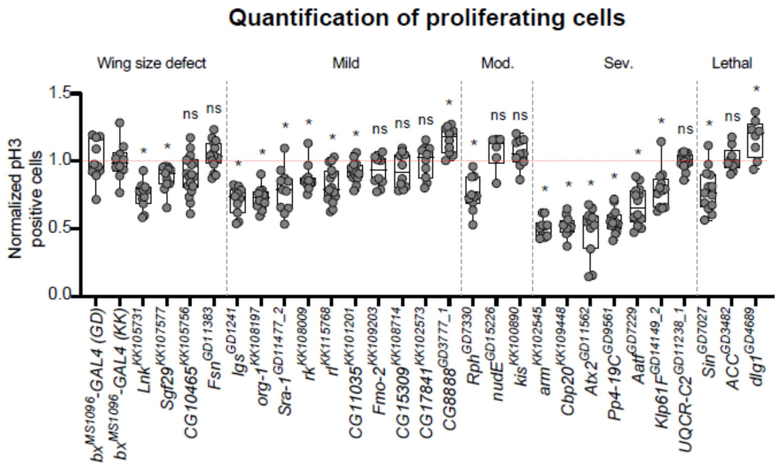

Figure R5. Box plots of dcp1-positive cells (top) and pH3-positive cells (bottom) in larval wing discs with knockdown of select fly homologs of CNV and neurodevelopmental genes, normalized to controls (n = 6–18, *p < 0.05, two-tailed Mann–Whitney test with Benjamini-Hochberg correction). We note that several RNAi lines showed severe dcp1 staining across the entire wing disc and could not be quantified. The number of dcp1 positive cells were calculated manually, and the number of pH3 positive cells were calculated using the AnalyzeParticles function in ImageJ. All boxplots are organized by adult wing phenotype (wing size defect, mild, moderate (mod.), severe (sev.), lethal). Boxplots indicate median (center line), 25th and 75th percentiles (bounds of box), and minimum and maximum (whiskers), with red dotted lines representing the control median.

8. ***In the introduction (line 119) you state you observed 79 genes. I think it should be done similar to figure 1 legend "We evaluated 59 Drosophila homologs of genes within 10 CNV regions and 20 known neurodevelopmental genes (79 total homologs)." This makes it clear what genes your looking at. Sometimes referring to 79 genes and then sometimes referring it as 59 genes +20 others made it unclear to follow the number of genes that was observed. Having it clear in the intro would help.***

Response: We thank the reviewer for the helpful suggestion, and have modified the Introduction as follows:

> ***Introduction, page 6**: "In this study, we tested tissue-specific and cellular phenotypes of 79 total fly homologs, including 59 fly homologs of human genes within ten pathogenic CNV regions and 20 genes associated with neurodevelopmental disorders."*

9. ***Also it is not clearly stated that the 20 neurodevelopment genes are CNV genes or not.***

Response: We selected the 20 neurodevelopmental genes based on curation of large-scale sequencing studies of neurodevelopmental disorders (Fromer et al., 2012, 2014; Gilissen et al., 2014; Iossifov et al., 2014; Martin et al., 2014), conserved functional roles in model organisms (Luo et al., 2012; De Rubeis et al., 2014), presence of fly homologs based on DIOPT scores, and availability of RNAi stocks (Iyer et al., 2016). These genes are not within rare pathogenic CNV regions, with the exception of *TBX1* (*org-1*), within the 22q11.2 deletion associated with DiGeorge syndrome, and *UBE3A* (*Ube3a*), within the Prader-Willi/Angelman syndrome region. As both of these genes are known causative genes for developmental phenotypes, we grouped these genes with the other neurodevelopmental-associated genes.

> ***Methods, page 23:** "We note that 2/20 neurodevelopmental genes, TBX1 (22q11.2 deletion) [92] and UBE3A (Prader-Willi/Angelman syndrome region) [93], are located within other pathogenic CNV regions."*

REFERENCES

Bellen, H.J., Tong, C., and Tsuda, H. (2010). 100 years of Drosophila research and its impact on vertebrate neuroscience: A history lesson for the future. Nat. Rev. Neurosci. *11*, 514–522.

Bier, E. (2005). Drosophila, the golden bug, emerges as a tool for human genetics. Nat. Rev. Genet. *6*, 9–23.

Bilen, J., and Bonini, N.M. (2007). Genome-wide screen for modifiers of ataxin-3 neurodegeneration in Drosophila. PLoS Genet. *3*, 1950–1964.

Bischof, J., Björklund, M., Furger, E., Schertel, C., Taipale, J., and Basler, K. (2012). A versatile platform for creating a comprehensive UAS-ORFeome library in Drosophila. Development *140*, 2434–2442.

Capdevila, J., and Guerrero, I. (1994). Targeted expression of the signaling molecule decapentaplegic induces pattern duplications and growth alterations in Drosophila wings. EMBO J. *13*, 4459–4468.

Cukier, H.N., Perez, A.M., Collins, A.L., Zhou, Z., Zoghbi, H.Y., and Botas, J. (2008). Genetic modifiers of MeCP2 function in Drosophila. PLoS Genet. *4*, e1000179.

Dietzl, G., Chen, D., Schnorrer, F., Su, K.-C., Barinova, Y., Fellner, M., Gasser, B., Kinsey, K., Oppel, S., Scheiblauer, S., et al. (2007). A genome-wide transgenic RNAi library for conditional gene inactivation in Drosophila. Nature *448*, 151–156.

Ewart, A.K., Morris, C.A., Ensing, G.J., Loker, J., Moore, C., Leppert, M., and Keating, M. (1993). A human vascular disorder, supravalvular aortic stenosis, maps to chromosome 7. Proc. Natl. Acad. Sci. U. S. A. *90*, 3226–3230.

Firth, H. V., Richards, S.M., Bevan, A.P., Clayton, S., Corpas, M., Rajan, D., Vooren, S. Van, Moreau, Y., Pettett, R.M., and Carter, N.P. (2009). DECIPHER: Database of Chromosomal Imbalance and Phenotype in Humans Using Ensembl Resources. Am. J. Hum. Genet. *84*, 524–533.

Fromer, M., Moran, J.L., Chambert, K., Banks, E., Bergen, S.E., Ruderfer, D.M., Handsaker, R.E., McCarroll, S.A., O'Donovan, M.C., Owen, M.J., et al. (2012). Discovery and statistical genotyping of copy-number variation from whole-exome sequencing depth. Am. J. Hum. Genet. *91*, 597–607.

Fromer, M., Pocklington, A.J., Kavanagh, D.H., Williams, H.J., Dwyer, S., Gormley, P., Georgieva, L., Rees, E., Palta, P., Ruderfer, D.M., et al. (2014). De novo mutations in schizophrenia implicate synaptic networks. Nature *506*, 179–184.

cGilissen, C., Hehir-Kwa, J.Y., Thung, D.T., Van De Vorst, M., Van Bon, B.W.M., Willemsen, M.H., Kwint, M., Janssen, I.M., Hoischen, A., Schenck, A., et al. (2014). Genome sequencing identifies major causes of severe intellectual disability. Nature *511*, 344–347.

Girirajan, S., and Eichler, E.E. (2010). Phenotypic variability and genetic susceptibility to genomic disorders. Hum. Mol. Genet. *19*, R176-87.

Girirajan, S., Rosenfeld, J.A., Coe, B.P., Parikh, S., Friedman, N., Goldstein, A., Filipink, R.A., McConnell, J.S., Angle, B., Meschino, W.S., et al. (2012). Phenotypic Heterogeneity of Genomic Disorders and Rare Copy-Number Variants. N. Engl. J. Med. *367*, 1321–1331.

Green, E.W., Fedele, G., Giorgini, F., and Kyriacou, C.P. (2014). A Drosophila RNAi collection is subject to dominant phenotypic effects. Nat. Methods *11*, 222–223.

Grossman, T.R., Gamliel, A., Wessells, R.J., Taghli-Lamallem, O., Jepsen, K., Ocorr, K., Korenberg, J.R., Peterson, K.L., Rosenfeld, M.G., Bodmer, R., et al. (2011). Over-Expression of DSCAM and COL6A2 Cooperatively Generates Congenital Heart Defects. PLoS Genet. *7*, e1002344.

Hu, Y., Flockhart, I., Vinayagam, A., Bergwitz, C., Berger, B., Perrimon, N., and Mohr, S.E. (2011). An integrative approach to ortholog prediction for disease-focused and other functional studies. BMC Bioinformatics *12*, 357.

Iossifov, I., O'Roak, B.J., Sanders, S.J., Ronemus, M., Krumm, N., Levy, D., Stessman, H.A., Witherspoon, K.T., Vives, L., Patterson, K.E., et al. (2014). The contribution of de novo coding mutations to autism spectrum disorder. Nature *515*, 216–221.

Itsara, A., Cooper, G.M., Baker, C., Girirajan, S., Li, J., Absher, D., Krauss, R.M., Myers, R.M., Ridker, P.M., Chasman, D.I., et al. (2008). Population analysis of large copy number variants and hotspots of human genetic disease. Am. J. Hum. Genet. *84*, 148–161.

Iyer, J., Wang, Q., Le, T., Pizzo, L., Grönke, S., Ambegaokar, S.S., Imai, Y., Srivastava, A., Troisí, B.L., Mardon, G., et al. (2016). Quantitative Assessment of Eye Phenotypes for Functional Genetic Studies Using Drosophila melanogaster. G3 (Bethesda). *6*, 1427–1437.

Iyer, J., Singh, M.D., Jensen, M., Patel, P., Pizzo, L., Huber, E., Koerselman, H., Weiner, A.T., Lepanto, P., Vadodaria, K., et al. (2018). Pervasive genetic interactions modulate neurodevelopmental defects of the autism-associated 16p11.2 deletion in Drosophila melanogaster. Nat. Commun. *9*, 2548.

Jackson, G.R., Salecker, I., Dong, X., Yao, X., Arnheim, N., Faber, P.W., MacDonald, M.E., and Zipursky, S.L. (1998). Polyglutamine-expanded human huntingtin transgenes induce degeneration of Drosophila photoreceptor neurons. Neuron *21*, 633–642.

Jacobsen, T.L., Cain, D., Paul, L., Justiniano, S., Alli, A., Mullins, J.S., Chun, P.W., Butchar, J.P., and Simcox, A. (2006). Functional analysis of genes differentially expressed in the Drosophila wing disc: Role of transcripts enriched in the wing region. Genetics *174*, 1973–1982.

Ji, X., Kember, R.L., Brown, C.D., and Bućan, M. (2016). Increased burden of deleterious variants in essential genes in autism spectrum disorder. Proc. Natl. Acad. Sci. U. S. A. *113*, 15054–15059.

Kaminsky, E.B., Kaul, V., Paschall, J., Church, D.M., Bunke, B., Kunig, D., Moreno-De-Luca, D., Moreno-De-Luca, A., Mulle, J.G., Warren, S.T., et al. (2011). An evidence-based approach to establish the functional and clinical significance of copy number variants in intellectual and developmental disabilities. Genet. Med. *13*, 777–784.

Kumar, J.P. (2012). Building an ommatidium one cell at a time. Dev. Dyn. *241*, 136–149.

Lek, M., Karczewski, K.J., Minikel, E. V, Samocha, K.E., Banks, E., Fennell, T., O'Donnell-Luria, A.H., Ware, J.S., Hill, A.J., Cummings, B.B., et al. (2016). Analysis of protein-coding genetic variation in 60,706 humans. Nature *536*, 285–291.

Luo, R., Sanders, S.J., Tian, Y., Voineagu, I., Huang, N., Chu, S.H., Klei, L., Cai, C., Ou, J., Lowe, J.K., et al. (2012). Genome-wide transcriptome profiling reveals the functional impact of rare de novo and recurrent CNVs in autism spectrum disorders. Am. J. Hum. Genet. *91*, 38–55.

Marquez, R.M., Singer, M.A., Takaesu, N.T., Waldrip, W.R., Kraytsberg, Y., and Newfeld, S.J. (2001). Transgenic analysis of the Smad family of TGF-beta signal transducers in Drosophila melanogaster suggests new roles and new interactions between family members. Genetics *157*, 1639–1648.

Martin, H.C., Kim, G.E., Pagnamenta, A.T., Murakami, Y., Carvill, G.L., Meyer, E., Copley, R.R., Rimmer, A., Barcia, G., Fleming, M.R., et al. (2014). Clinical whole-genome sequencing in severe early-onset epilepsy reveals new genes and improves molecular diagnosis. Hum. Mol. Genet. *23*, 3200–3211.

Morris, E.J., Michaud, W.A., Ji, J.-Y., Moon, N.-S., Rocco, J.W., and Dyson, N.J. (2006). Functional Identification of Api5 as a Suppressor of E2F-Dependent Apoptosis In Vivo. PLoS Genet. *2*, e196.

Nériec, N., and Desplan, C. (2016). From the Eye to the Brain. Development of the Drosophila Visual System. Curr. Top. Dev. Biol. *116*, 247–271.

Neumann, C.J., and Cohen, S.M. (1996). Distinct mitogenic and cell fate specification functions of wingless in different regions of the wing. Development *122*, 1781–1789.

Pizzo, L., Jensen, M., Polyak, A., Rosenfeld, J.A., Mannik, K., Krishnan, A., McCready, E., Pichon, O., Le Caignec, C., Van Dijck, A., et al. (2019). Rare variants in the genetic background modulate cognitive and developmental phenotypes in individuals carrying disease-associated variants. Genet. Med. *21*, 816–825.

Ponnam, S.P.G., Ramesha, K., Tejwani, S., Ramamurthy, B., and Kannabiran, C. (2007). Mutation of the gap junction protein alpha 8 (GJA8) gene causes autosomal recessive cataract. J. Med. Genet. *44*, e85.

Richards, A.A., and Garg, V. (2010). Genetics of Congenital Heart Disease. Curr. Cardiol. Rev. *6*, 91–97.

De Rubeis, S., He, X., Goldberg, A.P., Poultney, C.S., Samocha, K., Cicek, A.E., Kou, Y., Liu,

L., Fromer, M., Walker, S., et al. (2014). Synaptic, transcriptional and chromatin genes disrupted in autism. Nature *515*, 209–215.

Shulman, J.M., Imboywa, S., Giagtzoglou, N., Powers, M.P., Hu, Y., Devenport, D., Chipendo, P., Chibnik, L.B., Diamond, A., Perrimon, N., et al. (2014). Functional screening in Drosophila identifies Alzheimer's disease susceptibility genes and implicates tau-mediated mechanisms. Hum. Mol. Genet. *23*, 870–877.

Singh, M.D., Jensen, M., Lasser, M., Huber, E., Yusuff, T., Pizzo, L., Lifschutz, B., Desai, I., Kubina, A., Yennawar, S., et al. (2020). NCBP2 modulates neurodevelopmental defects of the 3q29 deletion in Drosophila and Xenopus laevis models. PLOS Genet. *16*, e1008590.

Soemedi, R., Topf, A., Wilson, I.J., Darlay, R., Rahman, T., Glen, E., Hall, D., Huang, N., Bentham, J., Bhattacharya, S., et al. (2012). Phenotype-specific effect of chromosome 1q21.1 rearrangements and GJA5 duplications in 2436 congenital heart disease patients and 6760 controls. Hum. Mol. Genet. *21*, 1513–1520.

Straub, J., Gregor, A., Sauerer, T., Fliedner, A., Distel, L., Suchy, C., Ekici, A.B., Ferrazzi, F., and Zweier, C. (2020). Genetic interaction screen for severe neurodevelopmental disorders reveals a functional link between Ube3a and Mef2 in Drosophila melanogaster. Sci. Rep. *10*, 1–12.

Thurmond, J., Goodman, J.L., Strelets, V.B., Attrill, H., Gramates, L.S., Marygold, S.J., Matthews, B.B., Millburn, G., Antonazzo, G., Trovisco, V., et al. (2019). FlyBase 2.0: The next generation. Nucleic Acids Res. *47*, D759–D765.

Twigg, S.R.F., and Wilkie, A.O.M. (2015). New insights into craniofacial malformations. Hum. Mol. Genet. *24*, R50–R59.

Vinayagam, A., Gibson, T.E., Lee, H.J., Yilmazel, B., Roesel, C., Hu, Y., Kwon, Y., Sharma, A., Liu, Y.Y., Perrimon, N., et al. (2016). Controllability analysis of the directed human protein interaction network identifies disease genes and drug targets. Proc. Natl. Acad. Sci. U. S. A. *113*, 4976–4981.

Vissers, J.H.A., Manning, S.A., Kulkarni, A., and Harvey, K.F. (2016). A Drosophila RNAi library modulates Hippo pathway-dependent tissue growth. Nat. Commun. *7*, 10368.

Wangler, M.F., Yamamoto, S., and Bellen, H.J. (2015). Fruit flies in biomedical research. Genetics *199*, 639–653.

Wangler, M.F., Hu, Y., and Shulman, J.M. (2017). Drosophila and genome-wide association studies: A review and resource for the functional dissection of human complex traits. Dis. Model. Mech. *10*, 77–88.

Wittmann, C.W., Wszolek, M.F., Shulman, J.M., Salvaterra, P.M., Lewis, J., Hutton, M., and Feany, M.B. (2001). Tauopathy in Drosophila: Neurodegeneration without neurofibrillary tangles. Science *293*, 711–714.

Zhu, S., Cordner, Z.A., Xiong, J., Chiu, C.T., Artola, A., Zuo, Y., Nelson, A.D., Kim, T.Y., Zaika, N., Woolums, B.M., et al. (2017). Genetic disruption of ankyrin-G in adult mouse forebrain causes cortical synapse alteration and behavior reminiscent of bipolar disorder. Proc. Natl. Acad. Sci. U. S. A. *114*, 10479–10484.
